# The social foundations for re-solving herbicide resistance in Canterbury, New Zealand

**DOI:** 10.1371/journal.pone.0286515

**Published:** 2023-06-02

**Authors:** Martin Espig, Roxanne J. T. Henwood

**Affiliations:** 1 AgResearch, Lincoln, Canterbury, New Zealand; 2 M.E. Consulting, Christchurch, Canterbury, New Zealand; Canakkale Onsekiz Mart University, TURKEY

## Abstract

Synthetic herbicides have revolutionised agricultural weed control. Herbicide resistance (HR) is a natural process through which weeds evolve to be no longer susceptible to a herbicide. Repeated use of similar herbicides can lead to the proliferation of resistant weed populations, with detrimental on-farm effects. To date, 267 weed species worldwide are resistant to at least one herbicide. Yet, achieving universal uptake of best practice principles to manage HR remains difficult. Historically not a high priority for New Zealand cropping farmers, resistance may be more prevalent than commonly assumed. This article contributes to emerging national management strategies and the international scholarship on the human dimensions of HR. Regarding resistance as a socio-biological challenge, we draw on qualitative social research with agricultural stakeholders in New Zealand’s main cropping region to outline important psychosocial preconditions for effective resistance management. Our findings show that these preconditions include: influencing awareness and attitudes, knowledge and skills; approaching HR as a shared responsibility; and supporting long-term and holistic thinking. We conclude that these preconditions form the social foundations for agricultural stakeholders’ capacity to enact best practice principles to continuously re-solve HR. This novel framing allows analytical differentiation between the capacity and ability to act, with practical recommendations and future research needing to address both components of effective HR management.

## 1. Introduction: Herbicide resistance in New Zealand

Synthetic herbicides are crucial for weed management in farm systems worldwide. Herbicides inhibit biological processes within susceptible plants through several metabolic mechanisms, their so-called mode of action, at target sites within that plant. All available herbicides function at one of 31 known sites of action, or a combination of several sites. First commercially released in the 1940s, herbicides helped to significantly enhance agricultural productivity and simplified weed control. Shortly after herbicides began to revolutionise weed management, instances of resistance started to emerge in the 1950s in the United States of America. Herbicide resistance (HR) refers to the evolution of targeted plant species to a point where they are no longer susceptible to a herbicide’s mode of action. To date, 267 weed species in 72 countries have been confirmed as resistant at 21 out of 31 known sites of action, including resistance to multiple modes of action [1]. HR poses major challenges for agricultural producers, including additional cost for alternative weed management and substantial yield or revenue losses [2–4].

Weed scientists and agricultural extension professionals have stressed for decades that farmers should reduce their reliance on a small range of herbicides and adopt best on-farm HR management recommendations. Key measures include the use of diverse weed control practices, incorporating non-synthetic controls, diversifying crop rotations, and applying different herbicide modes of action [3, 5–7]. While these measures do not stop individual plants evolving resistance traits, they can prevent the proliferation of resistant weed populations. Despite these efforts, uptake of best management practices is not universal and the number of confirmed instances of HR continues to increase worldwide [1].

In New Zealand, HR has historically not been regarded as a severe problem, due to relatively diverse crop and livestock rotations on many farms. Early cases of resistance were reported in the 1970s and their number has risen more rapidly over the last two decades [8, 9]. Across the country’s main grain cropping area on the Canterbury plains, HR has recently been confirmed in *Lolium perenne* (perennial ryegrass), *Avena fatua* (common wild oat), and *Lolium multiflorum* (Italian ryegrass) populations [9–12]. While it remains unclear how representative these cases are for resistance across New Zealand [8], random sampling on 20 per cent of cropping farms in one Canterbury district has identified resistant ryegrass populations on almost one third of sampled paddocks [13–15]. Resistance was also found among other weed species, which suggests that HR might be more prevalent in Canterbury and New Zealand than commonly assumed.

Despite HR becoming a growing challenge for New Zealand’s agricultural producers, industry-wide approaches to address HR are just starting to emerge. This paper contributes to both the development of these approaches in New Zealand and the growing body of international scholarship on the human dimensions of HR by presenting findings from qualitative social research with agricultural stakeholders in Canterbury. In regarding resistance as a socio-biological challenge that involves natural evolutionary processes and human management aspects, we examine the psychosocial preconditions for effective HR management. We conceptually frame these preconditions as the social foundations for re-solving resistance. Following an overview of social and cultural solutions to HR described in international scholarship in the next section, we propose that the outlined findings and conceptual framing add a novel analytical angle to the social research on the human dimensions of HR.

## 2. Social and cultural solutions to herbicide resistance

Weed scientists have explored the biophysical aspects of HR to better understand resistance mechanisms since first recorded instances in the 1950s [16, 17]. Not until more recently, however, has focused attention been given to the social and cultural aspects of weed management and HR. Less than a decade ago, Ervin and Jussaume [18 p407] remarked that “there has been virtually no empirical research that has collected information or analyzed the social factors that have contributed to the evolution of herbicide resistance.” They argue that this is problematic because successfully managing, and where possible avoiding, HR will involve a suite of social, cultural, economic, and political changes. Since this call to action, a growing body of scholarship has investigated the human dimensions of HR (e.g. [19, 20]).

Factors underlying prevalent herbicide use patterns in modern agricultural systems have been explored, especially those that may prevent changes to alternative practices, such as a strong reliance on synthetic herbicides due to them becoming progressively cheaper and easier to use [18, 21–23]. Other studies offer insights into how growers’ perceptions, beliefs, and fears in relation to HR can enable or obstruct potential solutions, for instance adopting integrated weed management practices [24, 25]. This research demonstrates that the interplay between a range of social, cultural, and biophysical drivers stabilises, and can lock in, established use patterns. Given the multifaceted social and biological characteristics of HR, some researchers thus regard its management as a socio-biological dilemma or wicked problem, which suggests that HR has no simple solutions due to the multitude of biological and technological drivers, complexities of human decision-making, and associated economic aspects [26–31]. In addition to well-established best practice recommendations, a range of multipronged solutions has, therefore, been proposed to address the various wicked-problem elements of HR.

Scholars have suggested that solutions need to involve the wider human dimensions of weed management, including economic and sociopolitical aspects [19, 28]. Following these perspectives, actions need to be taken at multiple levels, from local and regional actors, to national initiatives [32]; by individual farmers, farm communities, and the seed and chemical industries [33]; and at the wider system level [28]. Consequently, managing HR effectively must involve actors throughout the agricultural system, including farmers, advisors, scientists, regulators, policy makers, and agricultural supply companies [29]. Shaw and co-authors [34] suggest that bringing these stakeholders together should be accompanied by cohesive messages for how to manage HR. To provide more practical support and information to agricultural stakeholders, Doohan et al. [35] further highlight that more useful extension programmes could be established if weed scientists had better understandings of farmer beliefs and established two-way information exchanges with them. Weed scientists can act as intermediaries within the wider agricultural system by engaging in open dialogue with a range of actors and forming interdisciplinary partnerships to support meaningful stakeholder communication [32].

Other proposed solutions focus on on-farm management, HR-specific knowledge, and prevalent attitudes. These solutions highlight the need for shifting farmer mindsets and behaviours. Such changes may occur through farmers taking initiative to educate themselves about HR management, looking for weeds that survive herbicide applications, and taking a long-term strategic view [36, 37]. Other suggestions include farmers utilising farm performance data more effectively to support their weed management decisions [32]. Reflecting on how perceptions and behaviours may be changed, Moss [37] regards effective communication of information, a willingness to change, and the ability to change as crucial. Provision of information, different incentives, and regulation may all influence these factors. Attending to these prerequisites for enacting best management principles helps to better understand farmer perspectives and their ability to alter practices [37]. However, practice changes by individual farmers alone may be insufficient. Collective community action might be needed if resistant weeds are mobile across the landscape, either through natural causes (e.g., pollination) or anthropogenic factors (e.g., contaminated equipment) [38]. Some scholars have, therefore, argued for community-based approaches to establish effective weed control [21, 26, 39].

Another set of solutions includes regulatory measures and financial incentives to address economic and motivational barriers. Barrett et al. [40] argue that a combination of top-down regulation, voluntary industry regulation, and different incentives are likely to provide the best preconditions for HR management. However, there is disagreement on the role of regulation, with Shaw et al. [34] noting that disincentives (e.g., regulation) are not helpful when trying to frame resistance as a collective challenge. Powles and Gaines [7] describe how ‘regulatory creativity’ may provide alternative incentives, such as offering to extend product registration periods if chemical companies undertake stewardship actions like limiting recommended application frequencies of herbicides. Diverse voluntary and mandatory measures have thus been proposed in support of effective HR management.

Despite this suite of possible solutions, known instances of HR continue to increase in many agricultural systems around the world. This may be due to the proclaimed wicked-problem characteristics of HR, which suggests that proposed solutions face complex implementation barriers and can themselves create new challenges. It is then perhaps not surprising that well-established recommendations have often not led to corresponding actions by farmers and rural professionals. Therefore, critical questions remain as to what can prompt and enable agricultural stakeholders to better incorporate best practice principles into on-farm management. To address these questions, we present and then discuss a range of psychosocial preconditions for effectively managing resistance, which we argue form part of the social foundations for re-solving HR.

## 3. Materials and methods

### 3.1 Research location

**Fig 1:**
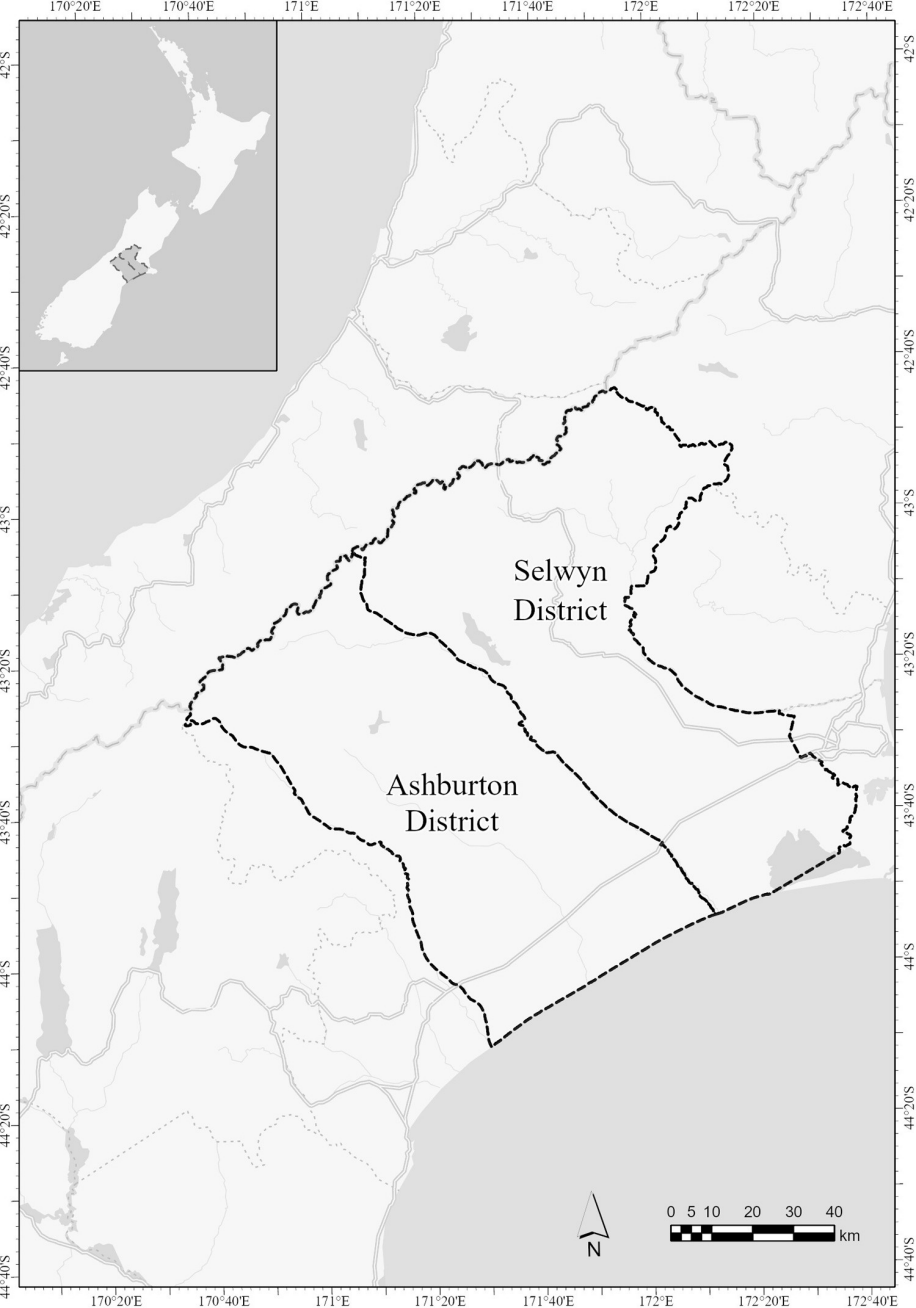
The Ashburton and Selwyn districts on New Zealand’s South Island. (source: created by Peter Pletnyakov based on Stats NZ’s data [43]).

Our research focused on a key crop growing area on New Zealand’s South Island, the Ashburton and Selwyn districts in the Canterbury region (see Fig 1), where the sector is more commonly referred to as arable farming. Canterbury has a population of around 600,000 people and approximately 82 per cent of residents are of European New Zealand ethnicity, which is more than 10 per cent above the New Zealand average. Those identifying as Māori (the country’s Indigenous people) represent 9.4 per cent, below the national average of 16.5 per cent. Other ethnic groups include Asian and Pacific peoples, which are similarly represented below the national average. The median age of Canterbury residents is higher than the national median age at 38.7 years. The region has some 7,000 farms covering over 2.5 million hectares of farm area, which is the second highest number of farms and largest farm area of all New Zealand regions [41, 42].

While the primary agricultural land uses in the region are livestock and dairy farming, Canterbury is New Zealand’s main region for grain production. Other prominent crops include certified seeds like ryegrass, clover, and brassica. Arable farmers in Canterbury usually run diverse rotations, including grains, seed crops, other crops (e.g., legumes, vegetable crops), and livestock production. Livestock production may include lamb and beef finishing, sheep breeding and raising, and dairy support (e.g., grazing dairy cows during their non-milking season). Crops are often contracted with buyers in advance, particularly seed crops.

Arable farmers commonly use different cultural controls to manage weeds, such as rotation of crops, cultivation, stubble burning, and fallowing. However, herbicides remain central to their systems, enabling certified seed crops to meet required standards for purity and allowing short durations between crops. They are often also seen as the most cost-effective weed control option. Potential restrictions on herbicide availability or use, whether through regulation or resistance, would thus have significant effects on farm systems and implications for viable crops.

### 3.2 Methods

This study is part of a five-year research programme that investigates the spread, mechanisms, controls, and potential solutions for HR. To identify associated social and cultural factors, qualitative social research through focus groups and interviews was conducted between November 2019 and June 2020. The study was approved by AgResearch’s Human Ethics Committee (#08/2019), and informed consent was recorded in written or verbal form prior to all research engagements. Empirical research was undertaken through two focus groups and eighteen qualitative interviews (see Table 1). One focus group was held with weed scientists and another with rural professionals (seed company agronomists, rural advisors, and representatives from industry support organisations). Focus groups included both male and female participants, with more males in the scientist group and approximately equal gender distribution among rural professionals. Both groups included participants in their early twenties to late sixties to include a spectrum of perspectives. The intent behind involving stakeholders from across the agricultural system was to account for the social and advisory networks involved in farmers’ decision-making processes related to weed management [31].

**Table 1:** Categories and number of participants involved in focus groups and interviews.

	Farmers and farm managers	Rural advisors and field agronomists	Crop protection industry representatives	Industry good bodies representatives	Weed scientist
1. FOCUS GROUPS	-	5	4	2	6
2. SEMI-STRUCTURED INTERVIEWS	12	3	-	-	3

Online interviews were subsequently conducted with twelve arable growers as well as a small number of weed scientists and rural advisors who did not participate in either focus group. Interview participants were purposively recruited through the authors’ network within the wider research programme and snowball sampling strategies. Approximately half of the growers had paddocks with resistant weeds, which had been identified through random HR sampling across the districts in 2019 [13–15]. Advisors and growers were all male, in their early twenties to sixties, and operated predominantly family-operated farms with a range of characteristics (e.g., size). While arable farm systems vary across Canterbury, grower interviews covered a typical spectrum of farm operations and farmer demographics in the Ashburton and Selwyn districts. This includes the common regional rotations described in the previous section, including cereal grains (e.g., wheat, oats, and barley), seed crops, and in many cases livestock components. Insights from the discussions with farm advisors and rural professionals further assisted in contextualising growers’ accounts and assessing their generalisability. Eighteen interviews were deemed sufficient once novel insights from new interviews became minimal and a saturation point was reached [44].

Nonetheless, several limitations are worth noting. First, the farm characteristics in both districts are not necessarily representative of other arable farm systems or farmer demographics across New Zealand, for instance maize growing regions on the country’s North Island. Furthermore, the arable industry is only one of several agricultural sectors (e.g., livestock and dairy farming). Second, while weed control frequently is part of male farmers’ duties within family-operated farms in this region, gender differences among farmers require further investigation. Third, additional research is needed to analyse in sufficient detail perspectives across agribusinesses operated by Māori, New Zealand’s Indigenous population.

Focus groups and interviews followed a semi-structured approach. Questions for participants were thus adjusted to the given setting, but they all focused on three thematic areas: HR knowledge and beliefs, stakeholders and other factors influencing decision-making around herbicide use, and solutions and barriers for HR management. Conversations were audio recorded, transcribed, and thematically analysed [45]. The primary author led the coding process with support from the secondary author, and both used the data analysis software NVivo to assist coding. The thematic analysis followed an inductive approach to elicit core themes on the drivers of farmers’ herbicide use (see [31]) and potential solutions to managing HR. Emerging themes were reviewed and refined through discussions between the authors and co-researchers, including with colleagues and partners within the wider research programme. The findings presented in the next section focus the thematic cluster around potential solutions to managing HR.

## 4. Results

Our empirical research identified three key principles for on-farm HR management: *diversity* of herbicide modes of action, which often require more diverse crop rotations; *timeliness* of weed control, including herbicide applications; and routinely *monitoring* weeds and the outcomes of weed control. Similar principles are well-documented internationally and have formed the basis for weed management recommendations for decades. Yet, these principles are not universally applied. Our research identified factors that influence adherence to these recommendations through an analysis of the drivers of herbicide use at the individual psychosocial, farm system, and socioeconomic system levels [31]. We present a summary of these interconnected drivers in the next section to highlight that weed control options, first and foremost, must be practical and profitable within existing farm systems. This overview provides the background for the second section that outlines empirical findings pertaining to the psychosocial preconditions for shifting weed management practices. In combination, these preconditions form the social foundations for continuously re-solving HR as a socio-biological issue. We then discuss the analytical and practical usefulness of this framing.

### 4.1 The drivers for herbicide use

Psychosocial drivers of herbicide use include agricultural stakeholders’ awareness and attitudes, knowledge and skills, motivation to change, and decision-making processes. While awareness of HR in New Zealand is seen to be generally increasing, we noticed considerable variation among farmers and rural professionals. Attitudes differed as well, with some growers feeling that the comparably high number of crops grown in rotation on many arable farms in the two districts prevents the development of resistance. Both farmers and rural professionals saw different levels of knowledge and skills related to best practice herbicide use among their peers, with many regarded as having low to moderate levels of HR-specific knowledge. Motivations for herbicide use include pecuniary and non-pecuniary aspects, such as farm profitability, yield maximisation, and practicality of weed control. In this context, herbicides are often seen as indispensable. Farmers highlighted that herbicide applications are but one factor in their daily decision-making, and that often multiple agronomists provide input into farm decisions. In the midst of this complexity, some growers are able to maintain long-term crop and herbicide strategies, but others rely on short-term advice that is focused on addressing specific situations.

Farm system level drivers of herbicide use include crop rotations, available technologies, natural biophysical factors, practicality, profitability, and conflicting priorities. Specific herbicides are often chosen depending on their suitability with a given crop and those that follow within seasonal rotations. Considerations to proactively avoid HR rarely determine what crops and associated herbicides are selected. Growers sometimes rely on spraying contractors, which limits their control over the process and timing. Additionally, lacking access to non-chemical weed control equipment can restrict options, such as specific tillage equipment. Natural factors like the timing for planting crops and a dependence on weather conditions also influenced herbicide use patterns. Practicality is a key driver, as weed management options need to fit into existing practices without adding further complexity or labour requirements. Profitability is also crucial and specific crops or weed management practices need to add value to a farm system and, in most cases, not add significant costs. Farmers noted that conflicting priorities can complicate herbicide use. For example, minimal cultivation systems to enhance soil health often depend on increased herbicide use, which potentially enhances HR risks.

Socioeconomic system level drivers of herbicide use include New Zealand’s crop contract system, agrichemical system, regulation, market requirements, and profitability. With many arable crops grown under contract in Canterbury, growers may be unable to strategically plan crop rotations across seasons, with implications for their ability to proactively rotate herbicide modes of action. Advice on herbicide options by agrichemical companies, either through sales or herbicide labels, significantly influences growers’ decisions. Rural professionals and growers also highlighted that regulation can restrict the use of certain herbicides or non-chemical weed control options. Domestic and export market requirements might similarly limit herbicide options or demand the rigorous use of specific herbicides, for instance to meet seed purity criteria. The combination of these socioeconomic system level drivers not only directly influences herbicide decision-making but also affects their farm profitability, which can then push some farmers towards more intensive operations and tighter crop rotations to maximise financial returns.

### 4.2 The social foundations for re-solving herbicide resistance

Based on the overview of these multifaceted practice drivers, this section outlines empirical findings regarding the social foundations for re-solving HR. We asked research participants a set of open-ended questions about what might support agricultural stakeholders in applying best management herbicide practices and which barriers to practice changes need to be overcome. We present a subset of these findings in four themes that capture core psychosocial preconditions for farmers and rural professionals to enact best practice principles.

#### 4.2.1 Raising awareness and changing attitudes

Weed scientists, rural professionals and several farmers stressed that agricultural stakeholders need to be aware that HR can become a major on-farm challenge and that being familiar with the core principles of best practice herbicide use is crucial. Diversity, timeliness, and monitoring were widely mentioned as core weed management principles to avoid the proliferation of resistant weeds. Rotating herbicide modes of action, routinely monitoring paddocks and swiftly removing plants surviving herbicide applications is critical for preventing weeds from reproducing. As one weed scientist emphasised:

good agronomic practices are really good. …. So, know what you sprayed and then know if the spray worked. And if it didn’t, being out there figuring out why it didn’t work and then not letting those plants go to seed no matter what. I think seed prevention in the field is number one because once you get a seed bank, those seeds stay in the soil for seven years or ten years ….

However, getting farmers and rural professionals’ attention to consider these steps before resistant weeds have become an on-farm issue was considered difficult. Furthermore, an only slowly changing attitude that existing crop rotations protect Canterbury farmers against HR was noted as a potential barrier to better awareness of HR risks. Many growers who were surveyed as having identified resistance on their farms saw the unexpectedly high percentage of positive results as raising ‘huge alarm bells’ and being a wake-up call [13–15].

Yet, openly discussing problems with resistance and potential management options was described as a major obstacle for the arable sector proactively working towards solutions. Several growers with identified resistant weeds on their farms overcame the social stigma associated with HR and attended a workshop organised by an arable industry body, with one farmer subsequently reflecting that he:

could see how someone wouldn’t want the neighbours to know. *…* I guess farming is a very closed book, a lot of neighbours don’t talk and it’s almost that shame thing. But … the few people that I talked to that were involved in the survey similarly felt the same way, I think, and it was actually good to be able to sit back and share it with each other.

Changing discourses around HR and removing the associated stigma of not being a ‘good farmer’ if cases of potential resistance emerge was seen as an important prerequisite for preventative measures and timely on-farm actions. As we outline below (see section 4.2.3), removing this stigma will likely require industry-wide dialogue and collaborative approaches between a range of agricultural stakeholders.

Raising awareness and changing attitudes is only a first step, though. Even if farmers are aware of best management practice recommendations, they may be unsure about how to translate them into practically and economically feasible changes to their specific farm system. Understanding the complexity behind each farm operation is thus crucial, as a weed scientist noted:

I think there’s a lot more difficulty in it, that each farm and each farmer is very unique and so you don’t have like ‘Oh, we know how you solve this problem, we’re just going to give you these three rules to follow and it’s all done, your resistance problems are fixed’.

For farmers and rural professionals, core principles and high-level recommendations can thus appear impractical if they are unable to turn them into suitable on-farm management practices or lack a support network that can assist them.

#### 4.2.2 Building knowledge and skills

In addition to raising awareness and familiarity with best practice principles, it is indispensable to further improve the industry-wide level of HR-related knowledge and skills. Building knowledge includes understanding how rotating different crops affects available herbicides, their different modes of action, and how complementary non-chemical control options can contribute to resistance management. Many rural professionals and farmers emphasised that location-specific knowledge and learning from practical experience are also important, for example by regularly walking paddocks to detect which weed control options work best under given circumstances.

Yet, most research participants mentioned that levels of formal and practical knowledge regarding weed control vary widely among growers, seed company field staff, and rural advisors. One weed scientist saw that, in the first instance, farmers need to improve their understanding of avoidance strategies, as they are the eventual on-farm decision makers:

I need to understand how resistance happens and what I can do to avoid it, which is essentially around rotating between mode of actions and having a diverse system so you can use a lot of different … mode of actions throughout a rotation order, no other ways of controlling weeds as well.

For other participants, all agricultural stakeholder groups need to build knowledge in this area. A rural professional with an international background regarded industry-wide education as crucial for continuously re-solving HR, including among his peers. For him, arable industry bodies should:

educate people on this subject, even just the ones willing to show up, is where it needs to start. We need to understand resistance and the rotation of chemical groups better, I find very often that fellow agronomists and advisors do not know the basics and have never seen a HRAC [Herbicide Resistance Action Committee] mode of action chart.

This sentiment was equally shared by many growers, most of whom acknowledged their own responsibilities but also the need for support from the wider arable industry. One farmer felt that:

there needs to be greater assistance in enabling farmers to learn more about this weed resistance and maybe some kind of assistance of… I don’t know whether it’s financial or certainly, probably, more educational.

Building HR-related knowledge and skills was thus seen by many participants as the responsibility of both farmers individually and the arable industry as a whole. In addition to the need to make agricultural stakeholders aware of resistance in the first place, cost and time commitments required for upskilling and learning were mentioned as potential barriers to an industry-wide increase in knowledge.

#### 4.2.3 Making herbicide resistance management a shared responsibility

Most participants saw that farmers are the key actors in HR management, given that they usually apply herbicides or instruct contractors. Some weed scientists and rural professionals noted that the first step is for growers to ‘own the problem’. Farmers were regarded to hold a range of responsibilities. For instance, one weed scientist noted that effective resistance management must start at the paddock level, so every farmer needed to regularly monitor each paddock and ensure suitable weed control. Several farmers also acknowledged that they saw it as their responsibility to share paddocks’ crop and herbicide histories with advisors to enable informed decision-making. This also includes responsibility for integrating subsequent advice into their overall farm system. As one grower explained, “it’s up to me to share that knowledge, you can’t expect say a [seed company representative], when they’re monitoring a paddock to have that total overview of your whole operation”. Others felt that farmers are responsible for seeking information and educating themselves around best practice herbicide use.

However, potential barriers for agricultural stakeholders proactively seeking to address HR were already outlined in the two preceding sections. These including lacking awareness due to herbicide use being but one of many on-farm management aspects, potentially conflicting priorities, and a social stigma associated with resistance. Removing the stigma around discussing resistance was identified as a critical component of re-solving associated challenges. This may involve changing the discourse from one of risk management to a forward-oriented notion of sustainable herbicide use, which might help to reduce negative connotations. Several farmers and rural professionals also highlighted the potential gains of sharing on-farm experiences and weed control problems with neighbours to remove the associated stigma and engage in farmer-to-farmer learning.

Making HR management a shared responsibility must also go beyond farmers. While growers are indeed oftentimes the final decision-makers, our research highlights the diverse factors and actors that influence their decisions, including multiple agronomists providing advice. Therefore, participants from across all stakeholder groups emphasised the need for collective action across the wider agricultural system. As one weed scientist remarked:

It’s a bit of a tragedy of the commons almost … because it is the farmer at an individual level that largely bears the cost and also has the responsibility, but it’s also … the wider community involved in farming that also needs to bear the responsibility as well.

A spectrum of responsibilities for the arable industry, agrichemical companies and weed scientists were mentioned in our research. These responsibilities range from sharing coherent key messages and standardised terminology related to HR management; establishing easy-to-use information resources to support farmers and rural professionals’ decision-making; offering real-time advice for specific weed control problems; or more research around specific risk scenarios (e.g., for individual herbicides and weed species) and novel control options. Many participants commented on the need to separate the herbicide recommendations of rural advisors from sales targets and would like trusted sources for independent advice, which may require pan-industry training programmes for rural professionals.

Industry-wide measures to support sustainable herbicide use would, on the one hand, be aimed at effectively managing resistance. On the other, several participants also emphasised the role of environmental regulation, market requirements and societal pressures as factors that can enable or prevent specific herbicide use patterns. One farmer commented that:

I see herbicide resistance has sort of got two sides to it. You’ve got the physical in the field aspect, but you’ve also got currently growing social pressures on what we do … in terms of our operation, pressure on chemical use is probably one of the biggest ones. That’s probably the biggest social pressure we face as an arable industry, that there’s more of a push on trying to go chemical free but how do you go chemical free if … we’ve got weeds ….

Steps towards re-solving HR are, therefore, crucial for practical and profitable on-farm weed management, but also for the New Zealand arable industry to respond to evolving societal perceptions of what constitutes responsible agricultural practices. Building knowledge and skills around diverse weed control options can then improve on-farm resilience in light of potential regulatory restrictions, such as banning of certain herbicides or non-chemical control options (e.g., crop residue burning). Conversely, reflecting on the role of socioeconomic system-level drivers demonstrates that responsibilities for sustainable herbicide use also sit with regulatory bodies. For example, many participants made suggestions for how herbicide labels could be improved and standardised to better highlight modes of action, which can support agricultural stakeholders in noticing repeated uses of the same herbicide group.

#### 4.2.4 Encourage long-term thinking and holistic approaches

Long-term planning of crop and herbicide rotations emerged as an important foundational aspect of sustainable herbicide use. For many participants, this involves a mindset shift away from reactionary or short-term weed control to proactive management strategies that increase diversity within a farm system as best as possible. However, planning crop and herbicide rotations across seasons can be challenging for Canterbury growers, given their reliance on a competitive crop contract system. As a rural professional remarked during a focus group:

the way farmers do business in New Zealand makes it very hard to do any kind of long-term planning. Planning is what I believe is key to using our current herbicides more sustainably. I think the answer is to influence the decision makers on farm, whether that’s the advisor, agronomist, or the farmer himself.

The crop contract system was not necessarily seen as a prohibitive obstacle to more strategic weed and herbicide management, though. Several rural professionals in the same focus group agreed that longer-term planning was still possible alongside these restrictions and other, potentially conflicting, priorities.

Keeping accurate and up-to-date records of herbicide usage was regarded as a critical prerequisite for such strategic planning. However, as a rural advisor criticised, “record keeping should be an absolute sitter but the amount of people that don’t keep records or don’t have a good record thing is ridiculous”. Among Canterbury arable farmers, only 20 per cent actively use the industry-developed ProductionWise software for crop record keeping, which includes a herbicide module. Others use handwritten records of herbicide applications. However, several participants noted that neither software nor hardcopy records are used for rotation planning in many cases, and that these could be utilised more effectively for HR management. This may include automated warning messages for repeated use of the same mode of action when using recording software. Establishing good record keeping practices is, therefore, an important foundation for HR management.

Long-term thinking and strategic decision-making then need to be complemented by holistic farm system approaches to weed management. Many participants, including farmers, reflected on the benefits of whole farm approaches that go beyond maximising short-term profitability to add long-term value to a farm system. In addition to adherence to fundamental agronomic principles, a range of important contributing factors were mentioned, such as timeliness of herbicide applications and attention to detail. A rural advisor emphasised the importance of these aspects:

I think as a whole farm approach, it’s the rotation, it’s the cleaning outside the crop, it’s … knowing that your grass weed that you’re going to target is present at the time and not too large and getting those application conditions 100%. So, you give everything … the best chance, you can’t leave anything hampered.

Other participants stressed that holistic approaches involve utilising all available weed management tools, including non-chemical control options where possible. For one weed scientist, it was thus:

no one particular thing, it really is just the integrated weed management model we’ve all been banging on about forever. That we need to use … all the tools and all the toolboxes.

Yet, several barriers to realising the potential benefits of farm system approaches and integrated weed management were mentioned. One limiting factor is the increased complexity involved in more diversified approaches. Building confidence with a range of chemical and non-chemical weed control options requires education and learning among agricultural stakeholders, particularly as widespread uptake of such integrated approaches is required. One grower, therefore, remarked:

We’ve got these new technologies which are wonderful, but are they practical or useable? Like is it just for … 5% of farmers? We’ve got to deal with the 80% of people … to adopt something, it has to be really adoptable and easy.

In addition to practicality, other participants highlighted regulatory restrictions and economic limitations, due to a need to maintain on-farm profitability, as potential barriers to diversified management approaches. One seed company representative suggested that these challenges could be addressed by investigating relevant economic ramifications through an industry-run, long-term systems trial.

Therefore, it is not only important to encourage long-term thinking and holistic approaches, but also to address associated barriers for enacting them within existing farm and agricultural systems. Overcoming barriers requires, in turn, an additional set of solutions that bring their own challenges, some of which have been described here. Combining the findings outlined across the four themes in this section demonstrates the multifaceted character of HR development and the interwoven steps required towards its (re)solution.

## 5. Discussion

Surveying the variety of psychosocial factors that are seen to contribute to re-solving HR offers a partial answer to Ward’s [19 p551] provocative question “why aren’t we doing it”, despite knowing what to do. Our findings demonstrate that lacking knowledge or technologies are often not the sole, or even main, barriers to the adoption of resistance management strategies, which is consistent with observations by other researchers [22, 24, 25, 32, 37]. Instead, various social and biological factors interact in ways that can make resistance an issue that resists simple solutions. The growing body of research that frames HR as a wicked problem, therefore, argues that all drivers must be understood and addressed [27, 28, 46]. Corresponding solutions must then affect behavioural and structural factors from the individual micro- to societal macro-level, including shifts in attitudes, norms, and beliefs [6, 28, 30, 36, 37]. The presented research with a range of agricultural stakeholders in New Zealand’s main crop growing region offers not only a novel geographic account for this body of research, but also presents several theoretical contributions of which we discuss three in more detail. In doing so, our analysis of the psychosocial preconditions for effective resistance management adds additional facets to the existing scholarship on the social and cultural foundations for addressing HR (e.g. [21, 33, 39]).

### 5.1 The need to continuously re-solve wicked problems

The framing of our presented empirical material directs attention to two core challenges of many wicked environmental management problems, namely that they are often not solved entirely and that partial solutions can face their own implementation barriers as well as create new challenges. Whereas it is widely recognised that addressing resistance requires multipronged approaches and potential trade-offs [27, 30, 33], many scholarly accounts are less explicit about HR management likely requiring continuous re-solutions for evolving challenges. This is due to several factors related to the socio-biological character of weed management, involving both natural evolutionary processes and societal influences on agricultural management [26].

On the one hand, resistance is an evolutionary process that involves regular genetic mutations in targeted species. Chosen weed control options, whether herbicidal or non-chemical, add selection pressures that may benefit specific mutations, which results in better chances for their survival and subsequent resistance to specific control options. This means that resistance is always going to be one aspect of weed management, which will demand that control options co-evolve. In addition to human-induced selection pressures, changing regional and global environmental conditions, such as climate change, also introduce pressures that can prompt weed evolution and the introduction of locally novel species. On the other hand, our analysis points to changing societal conditions in relation to agricultural management, such as environmental regulation, market requirements and societal expectations [31]. These factors will affect the demands placed on farm management and available weed control options. Therefore, understanding the psychosocial aspects we outlined as part of the social foundations for re-solving HR provides a basis for thinking more broadly about farmers and rural professionals’ underlying ability to continuously respond to these changing contexts.

### 5.2 The need to translate best management principles into local action

While scholars have noted that action is required by a range of actors on the local, regional, and inter-/national levels [28, 32], it is widely recognised that best management strategies must be tailored to local circumstances, such as farm system characteristics, and to the diverse characteristics of each weed species [33, 47]. Our research supports these calls, and we noted that translating best practice principles into practically and economically feasible local actions is a critical step. As such, weed management principles must be integrated into given farm systems and cannot follow a uniform approach. Farmers and actors in their rural support networks are the local experts who can, and should, lead this process of practical translation to enact key resistance management recommendations as best as possible. Doing so, however, requires support beyond information sharing and knowledge building, as our analysis demonstrates.

Framing our findings as psychosocial preconditions for subsequent action includes the shared responsibilities for effective resistance management among a range of actors as a critical component. Both individual cognitive and collective social aspects of HR are noted in the social scientific literature. In addition to well-established needs for farmers’ learning, scholars have stressed that collaborative approaches that involve close interaction between various agricultural stakeholders are required [22, 32, 35, 36, 39]. The notion of shared responsibilities as part of the social foundations for re-solving HR adds further understanding that collaboration is required, but that it does not automatically lead to intended outcomes. For these, other psychosocial preconditions we outlined also need to be established to improve agricultural stakeholders’ ability to enact changes. It is, therefore, important to also attend to the responsibilities of actors other than farmers in translating best management principles into local action and understand, among others, the perspectives of rural professionals and weed scientists.

### 5.3 The need to enhance the capacity to act

Thirdly, turning an ability to change into corresponding action goes beyond the social foundations we outlined and includes farm and agricultural system-level drivers that may enable or prevent practice changes. In this sense, we regard the outlined psychosocial preconditions as indispensable but, by themselves, insufficient for realising the ability to act. This understanding loosely draws on Stehr’s [48 p44] sociology of knowledge, wherein he contends that “knowledge is only a necessary, and not a sufficient, capacity for action”. The conceptualisation of knowledge as a capacity to act does, according to Stehr [48 p38], indicate “strongly that the material realization and implementation of knowledge are dependent on or embedded within the context of specific social, economic and intellectual conditions.” We would add that this equally involves biophysical conditions, especially in the case of HR management. Applying this perspective more broadly to the psychosocial preconditions we described, one can argue that building the social foundations for effective resistance management is crucial for enhancing agricultural stakeholders’ ability to act, but that realising corresponding actions then depends on a wider range of biophysical and social factors, for instance regulatory measures [7, 40].

Moss [37] offers a similar approach in that he distinguishes between the willingness and ability to change. Yet, under the latter Moss [37] combines drivers on the psychosocial (e.g., knowledge and skill), farm system (e.g., farm characteristics), and socioeconomic system (e.g., legal constraints) levels, which we regard as less helpful for focusing on the foundations for enhancing agricultural stakeholders’ ability to act upon herbicide use recommendations. Attending more specifically to the psychological, cognitive, social, and cultural factors that form the foundation for agricultural stakeholders’ capacity to act establishes the basis for analytically separating these from structural factors related to their ability to act. This analytical differentiation offers further explanatory potential to situations where agricultural stakeholders may be aware that changes in their weed management practices are required to reduce the reliance on a limited number of synthetic herbicides, but where they feel unable to do so due to structural constraints. Such perceived lock-ins as drivers for prevailing herbicide use patterns have been described in our research [31] and the international scholarship (e.g. [24, 25]). It is, therefore, important to simultaneously enhance actors’ awareness and willingness to change, for instance through outreach and extension activities, as well as to seek ways to enhance their capacity to enact corresponding practice changes within the context of broader systemic requirements.

## 6. Conclusion

Based on the analysis of presented empirical findings, we argued that successful resistance management requires to continuously re-solve associated challenges through practically and economically feasible on-farm actions. Enhancing farmers and rural professionals’ capacity to enact these practices as best as possible can be supported by fostering the psychosocial preconditions for effective resistance management. We summarised several of these preconditions under four overarching themes: (i) raising awareness and changing attitudes, (ii) building knowledge and skills, (iii) making HR a shared responsibility, and (iv) encouraging long-term thinking and holistic approaches. Following the conceptual framing of our findings as the social foundations for enacting best management principles, four corresponding practical steps to start re-solving HR as a socio-biological challenge can be proposed.

First, strengthening the social foundations for HR management involves holistically attending to the range of suggested solutions outlined above. The primary outcome is to build farmers and rural professionals’ capacity to enact best practice principles. Second, collaboration among a range of agricultural stakeholders is required to assess how the principles of ‘diversity, timeliness, and monitoring’ can be translated into feasible on-farm management practices, with growers and actors in their rural support network as key local experts. This will help to develop a range of practical and profitable best practice weed control options within their existing farm and socioeconomic systems. Third, farmers and rural professionals should seek ways to fully utilise the existing scope for action. This involves realising their current ability to act in the best possible manner. Fourth, all agricultural stakeholders can explore options for enhancing the scope for enacting best practice principles. The intended outcome is to improve farmers and rural professionals’ ability to act, for instance by advocating for reviews of regulatory measures that may prevent more diverse herbicide use.

The presented analysis primarily substantiates the first practical recommendation, thereby laying the conceptual groundwork for future research to address subsequent steps related to agricultural stakeholders’ ability to act upon best practice principles. For instance, our framing allows differentiation between the willingness to engage in long-term planning and the ability to do so in the context of a competitive seasonal crop contract system [31]. In contributing this analytical lens to the existing international scholarship, we follow Jordan and co-authors’ [49] call to broaden the problem definition around resistance management and to engage in more transdisciplinary research that takes a systems approach to weed control issues. As such, the presented analysis is a first step in establishing the social foundations for re-solving HR and demonstrates that the willingness, know-how, and ability to enact practice changes are all indispensable aspects of effective resistance management. Given the increasing significance of resistance globally and in New Zealand [1, 8, 9], this paper makes a timely contribution to the growing body of literature that seeks to systematically understand and influence the human dimensions of HR.
